# A 10-Year Retrospective Single-Center Study: Are Children With Cryptorchidism Operated On at the Recommended Age?

**DOI:** 10.7759/cureus.115206

**Published:** 2026-08-26

**Authors:** Nikolaos Baltogiannis, Fotini Fili, Melina E Baltogianni

**Affiliations:** 1 Pediatric Surgery, Children’s Hospital, Athens Medical Group, Athens, GRC; 2 Obstetrics and Gynecology, Agios Andreas Hospital, Patras, GRC

**Keywords:** congenital cryptorchidism, delayed orchiopexy, greece, pediatric surgery, testicular preservation, undescended testis

## Abstract

Background and aim

Spontaneous testicular descent after six months of age is uncommon, underscoring the importance of timely evaluation and surgical management of persistent cryptorchidism. This study aimed to evaluate the timing of surgical treatment for cryptorchidism at a tertiary pediatric surgical center in Athens, Greece, over a 10-year period and to assess adherence to contemporary recommendations for the timing of orchiopexy.

Methods

We retrospectively reviewed the medical records of 544 children who underwent surgical treatment for cryptorchidism during a 10-year period from January 2015 to December 2024. Patients were classified according to age at surgery into three groups: Group 1, ≤12 months; Group 2, 13-24 months; and Group 3, >24 months. Age at surgery, laterality, and operative approach were recorded.

Results

The mean age at surgery was 2.8 years. Right-sided cryptorchidism was present in 256 patients (47.1%), left-sided cryptorchidism in 196 (36%), and bilateral cryptorchidism in 92 (16.9%). Group 1 included 114 children (21%), Group 2 included 152 (27.9%), and Group 3 included 278 (51.1%). Nineteen patients (3.5%) underwent laparoscopic surgery because of an intra-abdominal testicular location.

Conclusions

Although the timing of orchiopexy appears to have improved compared with previous institutional experience, delayed treatment remains common. Only 21% of children underwent surgery during the first year of life, while 51.1% were treated after two years of age. These observations raise the possibility that delayed orchiopexy may represent a broader challenge within the Greek healthcare setting. Improved early recognition, timely referral, and closer adherence to guideline recommendations are warranted.

## Introduction

Cryptorchidism, or undescended testis, is among the most common congenital abnormalities of the male genital tract. Although spontaneous descent frequently occurs during the first months after birth, descent after six months of corrected age is uncommon. Persistent cryptorchidism beyond this period therefore warrants timely specialist evaluation and treatment [1-3].

The recommended age for orchiopexy has progressively decreased over recent decades as evidence has accumulated regarding the potential adverse effects of prolonged testicular malposition. Current European Association of Urology (EAU) guidelines recommend that treatment begin at six months of age, with orchiopexy ideally performed by 12 months and no later than 18 months [1]. Similarly, the American Urological Association (AUA) recommends that boys whose testes have not descended spontaneously by six months of corrected age undergo surgical treatment within the following year [2]. Guidance from the American Pediatric Surgical Association (APSA) likewise supports orchiopexy during infancy [3]. A systematic review by Chan et al. concluded that the available evidence supports orchiopexy during infancy, particularly between six and 12 months of age [4].

The rationale for early intervention is primarily related to preservation of testicular development and future fertility potential. Histological and volumetric studies have demonstrated more favorable testicular development following earlier orchiopexy [5]. A systematic review and meta-analysis by Allin et al. similarly found greater testicular volume and more favorable spermatogonial indices when orchiopexy was performed before one year of age [6]. Consequently, correction during infancy is now regarded as the standard approach.

Despite increasingly consistent recommendations, adherence to the recommended timing of surgery remains suboptimal in several healthcare systems. Williamson et al. demonstrated persistent failure to achieve orchiopexy within the recommended age despite publication of the AUA guideline [7]. Hensel et al., in a 10-year observational analysis of 3,587 cases, also demonstrated a considerable discrepancy between guideline recommendations and actual clinical practice [8]. Population-based analyses have similarly shown substantial variation in the timing of orchiopexy across healthcare systems [9].

Several potentially modifiable factors may contribute to delayed treatment. Delayed referral from primary care, missed diagnosis during routine examinations, limited parental awareness, geographic barriers, and healthcare-related factors have all been reported [10-14]. Jiang et al. specifically emphasized deficiencies in routine testicular examination and family education as potential contributors to delayed referral [10].

Evidence regarding the timing of orchiopexy in Greece remains limited. Kaselas et al. reported a retrospective series of 217 children treated for congenital cryptorchidism in Northern Greece. The median age at orchiopexy was 23 months, and delayed diagnosis and referral contributed to treatment outside the recommended age range [15].

## Materials and methods

Study design and setting

This retrospective, single-center observational study included children who underwent surgical treatment for cryptorchidism at the Second Department of Pediatric Surgery, “Aghia Sophia” Children’s Hospital, Athens, Greece, between January 2015 and December 2024.

Study population and patient selection

Patients were identified retrospectively from the department’s operative records and corresponding medical files. The study was intended to include the complete available surgical cohort during the defined study period rather than a sampled subset. Children were eligible for inclusion if they underwent surgical treatment for cryptorchidism during the study period and if the medical record contained sufficient information regarding age at surgery, laterality, and operative approach.

Cases with insufficient documentation to determine the relevant study variables were excluded from the analysis. Previously treated or recurrent cases were not included as new primary cases if the procedure represented reoperation for the same previously corrected condition.

Data collection

Data were extracted retrospectively from existing medical and operative records. The variables collected included age at surgical treatment, laterality of cryptorchidism, age category at the time of surgery, and laparoscopic treatment for an intra-abdominal testicular location. Patients were classified into three predefined age groups according to age at surgery: Group 1, ≤12 months; Group 2, 13-24 months; and Group 3, >24 months.

Because these historical age categories were used in the available dataset, the study was not designed to calculate the exact proportion of patients undergoing surgery before or after the current 18-month upper guideline threshold. Instead, the primary objective was to evaluate the timing of surgical treatment and to characterize the extent of delayed orchiopexy in relation to contemporary guideline recommendations.

Statistical analysis

The analysis was descriptive. Categorical variables were summarized as absolute frequencies and percentages, while age at surgery was expressed as the mean.

No multivariable analysis of predictors of delayed surgery was performed because potentially relevant explanatory variables, including age at initial diagnosis, referral interval, waiting-list duration, socioeconomic factors, geographic factors, and specific reasons for delayed treatment, were not consistently available in the historical records.

Ethical considerations

This study was based on a retrospective review of existing medical records. No additional diagnostic or therapeutic intervention was performed for the purposes of the study, and no identifying patient information is presented in the article.

## Results

A total of 544 children underwent surgical treatment for cryptorchidism between January 2015 and December 2024. The mean age at surgery was 2.8 years. Right-sided cryptorchidism was present in 256 patients (47.1%), left-sided cryptorchidism in 196 patients (36%), and bilateral cryptorchidism in 92 patients (16.9%). Regarding age at surgery, 114 children (21%) were operated on at ≤12 months of age, 152 (27.9%) between 13 and 24 months, and 278 (51.1%) after 24 months of age. Therefore, only approximately one in five children underwent surgical correction during the first year of life, whereas more than half underwent surgery after the age of two years. Nineteen patients (3.5%) underwent laparoscopic surgery because of an intra-abdominal testicular location.

The demographic and clinical characteristics of the study cohort are summarized in Table 1.

**Table 1:** Demographic and clinical characteristics of the study cohort.

Characteristic	Number of patients (n)	Percentage (%)
Total patients	544	100
Right-sided cryptorchidism	256	47.1
Left-sided cryptorchidism	196	36
Bilateral cryptorchidism	92	16.9
Surgery at ≤12 months	114	21
Surgery at 13-24 months	152	27.9
Surgery at >24 months	278	51.1
Laparoscopic surgery for intra-abdominal testis	19	3.5

## Discussion

The principal finding of this study is that a substantial proportion of children with cryptorchidism continue to undergo surgical treatment later than recommended. Among 544 children treated during the 10-year study period, 114 patients (21%) underwent surgery during the first year of life, whereas 278 patients (51.1%) underwent treatment after the age of two years. The mean age at surgery was 2.8 years. These findings contrast with contemporary European and North American recommendations supporting surgical correction during infancy [1-3]. The EAU recommends treatment beginning at six months, with orchiopexy ideally completed by 12 months and no later than 18 months [1]. The AUA similarly recommends surgical correction within one year after persistence of cryptorchidism at six months of corrected age [2]. APSA educational guidance likewise supports orchiopexy during infancy [3].

The rationale for early intervention is primarily related to preservation of testicular development and future fertility potential. Earlier orchiopexy has been associated with more favorable testicular growth and histological markers of future fertility [4-6]. Kollin et al. demonstrated improved testicular growth following orchiopexy performed at nine months compared with surgery at three years [5]. Similarly, the systematic review and meta-analysis by Allin et al. found greater testicular volume and more favorable spermatogonial indices when orchiopexy was performed before one year of age [6]. Although definitive long-term fertility outcomes are more difficult to establish, the available evidence supports avoiding unnecessary delay.

The persistence of delayed orchiopexy observed in our cohort is not unique to our institution. Similar findings have been reported internationally. Williamson et al. found that a substantial proportion of boys continued to undergo orchiopexy beyond the AUA-recommended 18-month target despite longstanding guideline publication [7]. Hensel et al., in a 10-year observational analysis of 3,587 cases, likewise demonstrated a considerable discrepancy between recommended and actual timing of surgery [8]. Population-based data from universal healthcare systems have also shown that, although the age at orchiopexy has generally decreased over time, substantial variation in adherence to recommended timing persists [9]. Delayed referral appears to be one of the most consistently reported modifiable contributors to late treatment. Jiang et al. identified delayed referral as an important factor and highlighted missed or inadequately documented testicular examinations and limited family education as possible contributors [10]. Sengar et al. similarly reported delayed referral and late recognition among children presenting for orchiopexy beyond the recommended age [11]. Comparable observations have also been reported in Austria and other European settings, where delayed referral persisted despite awareness of contemporary recommendations [12,14]. Geographic and healthcare access factors may also influence the timing of treatment, as suggested by studies comparing rural and urban populations [13].

These factors, however, were not systematically available in our historical medical records and therefore should not be interpreted as findings of the present study. Our analysis describes the timing of orchiopexy but does not establish the causes of delayed treatment or identify independent predictors of delay.

Our current findings suggest improvement compared with previous institutional experience. An earlier analysis from our department included 840 patients treated during 2005-2015 and was presented at the 53rd Panhellenic Pediatric Congress. In that cohort, the mean age at surgical treatment was 3.9 years; 77 children (9.2%) underwent surgery by 12 months of age, and 462 children (55%) were treated after 24 months. In the current cohort, 114 children (21%) underwent surgery by 12 months, 278 children (51.1%) were treated after 24 months, and the mean age at surgery decreased to 2.8 years. These historical comparisons should be interpreted cautiously because the earlier findings were presented as conference data and the two cohorts were not designed as a formal comparative longitudinal study. Nevertheless, they suggest gradual improvement in clinical practice while demonstrating that substantial delays remain.

Our findings are also consistent with published observations from another geographic region of Greece. Kaselas et al. studied 217 children undergoing orchiopexy in Northern Greece and reported a median operative age of 23 months, with delayed diagnosis and referral contributing to treatment outside the recommended age range [15]. Although the similarity between these geographically distinct single-center cohorts is noteworthy, the present study cannot establish whether delayed orchiopexy represents a nationwide healthcare problem. Rather, these observations raise a hypothesis that should be evaluated through multicenter or population-based studies.

This pattern mirrors the international literature. Although recommendations regarding early orchiopexy are widely disseminated, studies from North America and Europe continue to identify substantial delays between diagnosis, referral, and surgical correction [7-14]. The consistency of these findings suggests that publication of guidelines alone may be insufficient and that active measures aimed at improving implementation are required. Several practical steps could potentially improve the timing of treatment. Routine examination and accurate documentation of testicular position during infancy are fundamental. Persistent undescended testes should prompt timely referral to pediatric surgical or urological services after six months of corrected age. Improved communication between pediatricians and pediatric surgeons, standardized referral pathways, parental education, and minimization of unnecessary waiting periods may further reduce delays. These measures are supported by previously published literature on delayed referral and should be regarded as potential quality improvement strategies rather than conclusions derived directly from the present dataset [10-14].

Nineteen patients (3.5%) in our cohort underwent laparoscopic surgery because of an intra-abdominal location of the testis. Although operative technique was not the primary focus of this study, this subgroup represents children requiring laparoscopic evaluation and treatment of nonpalpable intra-abdominal testes.

Limitations

The present study has several important limitations. First, its retrospective design makes the analysis dependent on the completeness and accuracy of historical medical records. Second, the study was conducted at a single tertiary pediatric surgical referral center. This setting introduces the possibility of selection and referral bias, as patients referred to a tertiary center may not be representative of the broader pediatric population. Consequently, the findings should not be interpreted as population-based estimates, and their generalizability to other institutions or to the entire Greek healthcare system is limited.

In addition, the available historical records did not permit systematic evaluation of several potentially important determinants of delayed treatment, including age at initial diagnosis, interval from diagnosis to specialist referral, waiting-list duration, socioeconomic or geographic factors, family-related factors, and specific reasons for delayed surgery. Therefore, the present study can describe the timing of orchiopexy but cannot determine the causes of delayed treatment or identify independent predictors of delay.

Another limitation is that patients were historically categorized as ≤12 months, 13-24 months, and >24 months. These predefined categories do not permit precise calculation of the proportion undergoing surgery before or after the current 18-month upper guideline threshold. The results should therefore be interpreted as a descriptive assessment of surgical timing rather than a direct measurement of adherence to the 18-month recommendation.

Despite these limitations, the relatively large cohort and the 10-year study period provide useful real-world information regarding contemporary surgical practice and allow comparison with previous institutional and published Greek data.

## Conclusions

Delayed surgical treatment of cryptorchidism remains common in our institutional experience despite contemporary recommendations supporting orchiopexy during infancy. Although the timing of surgery appears to have improved compared with our previous institutional experience, substantial delays remain. Multicenter or population-based studies are needed before broader conclusions regarding national practice can be drawn. Improved early recognition, timely referral, and coordination between pediatricians and pediatric surgeons remain reasonable targets for improving the timing of treatment.
